# Efalizumab: results of a 3-year continuous dosing study for the long-term control of psoriasis

**DOI:** 10.1111/j.1365-2133.2008.08548.x

**Published:** 2008-05

**Authors:** C Leonardi, A Menter, T Hamilton, I Caro, B Xing, AB Gottlieb

**Affiliations:** Central Dermatology, Saint Louis University School of Medicine St Louis, MO 63117, U.S.A.; * Baylor Research Institute Dallas, TX, U.S.A.; † Atlanta Dermatology, Vein & Research Center, LLC Alpharetta, GA, U.S.A.; ‡ Genentech, Inc. South San Francisco, CA, U.S.A.; § Department of Dermatology, Tufts–New England Medical Center Boston, MA, U.S.A.

**Keywords:** efalizumab, heavy patient response, immunosuppressant, monoclonal antibody, plaque psoriasis, T-cell modulation

## Abstract

**Background:**

Efalizumab, a T-cell-targeted, recombinant, humanized, monoclonal IgG1 antibody, inhibits key T-cell-mediated steps in the pathogenesis of psoriasis. Efalizumab is approved for the treatment of moderate-to-severe chronic plaque psoriasis in adults in more than 50 countries.

**Objectives:**

To evaluate the efficacy and safety of long-term, continuous efalizumab therapy in patients with psoriasis.

**Methods:**

This open-label, multicentre phase III study enrolled 339 patients with moderate-to-severe chronic plaque psoriasis. During the initial 3-month phase, patients received subcutaneous efalizumab 2 mg kg^−1^ weekly with randomization to receive concomitant fluocinolone acetonide or placebo ointment during month 3. The second phase was a long-term observational period; patients achieving a ≥ 50% improvement in the Psoriasis Area and Severity Index (PASI) score were eligible to receive efalizumab 1 mg kg^−1^ weekly for up to 33 months. The final 3-month treatment period was an optional transition period for patients who completed the 33-month segment before efalizumab became commercially available.

**Results:**

After 3 months, 41·3% of patients achieved a ≥ 75% improvement in PASI (PASI-75) and 13·0% achieved a ≥ 90% improvement (PASI-90). Continued improvement was observed: 45·4% and 24·5% achieved PASI-75 and PASI-90, respectively, at the end of the observational phase. The safety profile was stable, with no new or no increase in common events over 36 months of treatment.

**Conclusions:**

This was the longest continuous study using a biologic therapy for psoriasis. Clinical benefit of efalizumab improved over the first 18 months and was maintained during 36 months of continuous therapy. Long-term efalizumab therapy is appropriate for many patients with plaque psoriasis.

**Conflicts of interest:**

C.L. with 3M Pharmaceuticals, Abbott, Allergan, Altana, Amgen, Astellas-Biogen, Bristol Myers, Centocor, CombinatoRx, Fujisawa Healthcare, Galderma, Genentech, Merck Serono International SA, Schering Plough, RTL, Vitae and Warner Chilcott; A.M. with 3M Pharmaceuticals, Abbott, Allergan, Allermed, Amgen, Astralis, Berlex, Biogen Idec, Celgene, Centocor, Cephalon, Collagenex Pharmaceuticals, CombinatoRx, Connetics, Corixa, Dermik Laboratories, Doak Dermatologics, Dow, Ferndale Laboratories, Fujisawa Healthcare, Galderma, Genentech, Genzyme, GlaxoSmithKline, Ligand Pharmaceuticals, Medicis, MedImmune, Novartis Pharmaceuticals, Otsuka Pharmaceutical, Protein Design Labs, QLT USA, Regeneration Pharma AG, Roche, Merck Serono International SA, Sinclair, Synta Pharma, Thermosurgery, Vertex, Warner Chilcott, Wyeth, XOMA and Zars; T.H. with Genentech; A.B.G. with Abbott, Actelion, Almirall, Amgen, Beiersdorf, Biogen Idec, Bristol Myers Squibb, Can-Fite, Celera, Celgene, Centocor, DermiPsor, Eisai, Genentech, Immune Control, Incyte, Kemia, Medacorp, Medarex, Novo Nordisk, Pharmacare, Roche, RxClinical, Sankyo, Schering Plough, TEVA, UCB, Warner Chilcott and Wyeth. All income derived from these sources goes to her employer. I.C. and B.X. are employees and stockholders of Genentech.

Psoriasis is a chronic autoimmune disorder requiring long-term treatment for symptom control.^1^ Systemic therapies, such as methotrexate, ciclosporin, retinoids, corticosteroids and phototherapy, are traditionally used to treat patients with moderate-to-severe disease. However, the long-term use of these therapies may be limited by inconvenience (phototherapy) or increased toxicities,^2^ which may include liver and bone marrow toxicity for methotrexate,^3^,^4^ renal toxicity and hypertension for ciclosporin,^5^,^6^ teratogenicity for retinoids,^4^,^7^ and an increased risk of skin cancer for phototherapy.^8^ Rotational, combination and sequential treatment strategies are employed to reduce toxicity, leading to cycles of disease remission and relapse, with a negative impact on patients’ quality of life.^3^,^9^–^11^ Efalizumab is a recombinant humanized monoclonal IgG1 antibody that targets key T-cell-mediated steps in the immunopathogenesis of psoriasis.^12^ Multiple clinical trials of 12–24 weeks’ duration have demonstrated the efficacy and safety profile of efalizumab and led to its approval in more than 50 countries for the treatment of adult patients with moderate-to-severe chronic plaque psoriasis.^13^–^17^

Psoriasis is a chronic disease requiring long-term therapy. A placebo-controlled study of this length would raise ethical and retention concerns. The use of a systemic comparator arm (e.g. ciclosporin or methotrexate) for this length of time is also problematic because of cumulative toxicity and blinding. This 36-month (3-year) continuous dosing study of efalizumab is the longest prospective study of a biologic therapy in psoriasis to date.

## Materials and methods

### Study design

**Fig 1:**
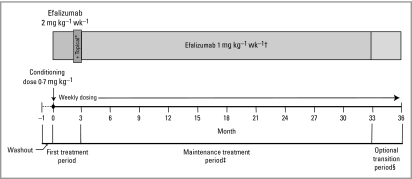
Study design. *All patients continued to receive efalizumab during weeks 9–12; patients were also randomized to receive either topical placebo or topical steroid; the final efalizumab dose in this 3-month (12-week) first treatment period was administered at week 11. †Through month 15, patients who relapsed ended participation in the current 3-month segment and started the next segment at 2 mg kg^−1^ weekly with an option to increase to maximum dosage of 4 mg kg^−1^ weekly for up to 4 weeks following relapse. After month 15, no increase in dose was permitted. ‡Patients who achieved a ≥ 50% improvement in the Psoriasis Area and Severity Index score or static Physician's Global Assessment of mild, minimal or clear at the end of the first treatment period were eligible to enter the maintenance treatment period. §The optional transition period was made available to patients who had completed 33 months of efalizumab therapy prior to the availability of commercial drug. Other patients transitioned to commercial efalizumab after month 33.

This was an open-label, multicentre, phase III clinical study. Details of the study design have also been previously described^18^,^19^ (Fig. 1). Patients were adults (aged ≥ 18 years) with a diagnosis of psoriasis for ≥ 6 months, with a Psoriasis Area and Severity Index (PASI) score ≥ 12 and psoriasis covering ≥ 10% of body surface area (BSA), who were candidates for systemic therapy. All investigator sites received Institutional Review Board approval prior to initiating the study, and all patients provided signed informed consent.

The aims of the study were to evaluate the duration of response and the effectiveness of prolonged maintenance therapy as reflected by the PASI score during long-term treatment with efalizumab. Previous clinical studies have indicated that some patients respond with relatively long-lasting residual erythema in sites of resolving plaque psoriasis during efalizumab treatment. To address the erythema, topical therapy was introduced during the third month of treatment after efalizumab typically had effected an improvement in total disease burden. Thus, an early endpoint of this study was a comparison of the efficacy of 12 weekly injections of efalizumab with and without concomitant administration of topical corticosteroid ointment added during the third month of the treatment period.

During the first 3 months (12 weeks), the initial phase of the study, patients received subcutaneous (SC) efalizumab 2 mg kg^−1^ weekly. To address the concern that persistent erythema would influence PASI responses and hinder patient entry to the second phase of the study, the effect of concomitant use of a weak topical steroid was investigated during month 3. Patients were randomized to receive concomitant fluocinolone acetonide ointment 0·025% (*n* = 169) or placebo equivalent (white petrolatum ointment; *n* = 170) during weeks 9–12, as described previously.^18^ The second phase of the study was a long-term observational period in which after 3 months, patients who achieved a ≥ 50% improvement in PASI relative to baseline (PASI-50) or a static Physician's Global Assessment (sPGA) rating of mild, minimal or clear on the Overall Lesion Severity (OLS) scale were eligible for an additional period of up to 12 months of maintenance therapy in 3-month segments with SC efalizumab 1 mg kg^−1^ weekly. Patients who did not achieve the required level of clinical benefit were withdrawn from the study. Subsequent amendments to the protocol extended the study so that patients were eligible for up to a total of 33 months of therapy. If patients experienced a protocol-defined relapse (loss of ≥ 50% of the PASI improvement achieved between baseline and week 12) during months 4–15, they immediately ended participation in their current 3-month segment and started their next segment at an escalated dosage of 2 mg kg^−1^ weekly. At the investigator's discretion, efalizumab could be escalated up to 4 mg kg^−1^ weekly. In total, 25 patients were treated with an escalated dose of 3 mg kg^−1^ weekly (*n* = 10) for a median duration of 4 weeks (range 2–55) or 4 mg kg^−1^ weekly (*n* = 15) for a median duration of 4 weeks (range 2–21). However, dose escalation was not permitted after month 15 per protocol. Subsequent analysis of data from multiple clinical trials confirmed the dosage of 1 mg kg^−1^ weekly as the appropriate regimen for further studies in psoriasis.^13^,^14^,^20^ The final 3-month treatment segment (months 34–36) was a transition period to bridge eligible patients from the study to commercial efalizumab or another appropriate therapy, depending upon the patient's date of completion of month 33 relative to the availability of commercial drug.

Better to reflect treatment patterns in clinical practice, selected concomitant psoriasis medications were allowed during the maintenance and transition treatment periods, including emollients, scalp preparations, topical therapies, or ultraviolet (UV) B phototherapy. All systemic psoriasis therapies were excluded 1 month prior to the first dose of efalizumab and throughout the study.

### Efficacy assessment and analysis

The study was divided into 3-month (12-week) segments, and PASI responses were evaluated at month 3 on the last day of each treatment segment. The intent-to-treat (ITT) analysis involved all 339 patients who entered into the study. Patients who discontinued treatment during the first treatment period (months 0–3) were classified as nonresponders, and patients who did not achieve a PASI-50 or an sPGA score of mild, minimal or clear within the first treatment period were discontinued from the study and considered nonresponders for the remainder of the study. If a patient discontinued during the maintenance treatment period (months 4–33), his or her last PASI score was carried forward for each successive time point (last observation carried forward, LOCF). Patients who received an excluded systemic psoriasis therapy for a psoriasis indication were considered nonresponders for the segment in which they first received the excluded medication and in all subsequent segments. Patients who received a systemic psoriasis medication for an indication other than psoriasis had their last PASI score prior to the segment in which they first used the systemic therapy carried forward for all subsequent segments (LOCF).

Secondary to the ITT analysis, an as-treated analysis included only those patients who remained in the study and initiated each treatment segment. Although not as conservative as the ITT–LOCF analysis method, as-treated analysis is reflective of the efficacy seen in the patients who persist on therapy over time.

In a separate ITT analysis of patients by weight, patients were divided into two groups, heavy (≥ 91 kg, *n* = 173) and nonheavy (< 91 kg, *n* = 166) patients, and the analysis was performed on each weight group with LOCF for missing values.

As is common in long-term studies, this study had a number of patient discontinuations. Given that there is currently no universally applicable method for handling missing values [as recognized by International Conference on Harmonisation (ICH) guidelines and the Committee for Proprietary Medicinal Products (CPMP)],^21^,^22^ the effect of continuous efalizumab therapy on PASI score was analysed using two methodologies (as-treated and ITT) to provide a reliable range of estimates of efficacy.

### Safety assessment and analysis

To evaluate safety, the incidence of adverse events was analysed during each 3-month treatment segment. Coding Symbols for Thesaurus of Adverse Reaction Terms (COSTART) definitions were used. Safety assessments were conducted using the as-treated population. All patients who received a concomitant systemic medication were included in the safety analyses, regardless of the indication.

### Role of the funding source

Genentech, Inc. participated in study design, data collection and data analysis; and Genentech, Inc. and Merck Serono International SA participated in the writing of this report. The corresponding author had full access to all the data in the study and had final responsibility for the decision to submit for publication.

## Results

### Baseline characteristics

A total of 339 patients with moderate-to-severe psoriasis at baseline enrolled in the study. The first patient enrolled on 2 February 2001, and the last patient enrolled on 18 July 2001. The patient population exhibited a median PASI score of 17·2 (mean ± SD 19·8 ± 8·3) with a median BSA of 25·0% (mean ± SD 31·5 ± 18·5) and a median duration of 16·0 years (mean ± SD 17·9 ± 10·6). Patient demographics and detailed baseline characteristics have been reported previously.^18^,^19^

### Patient disposition

**Fig 2:**
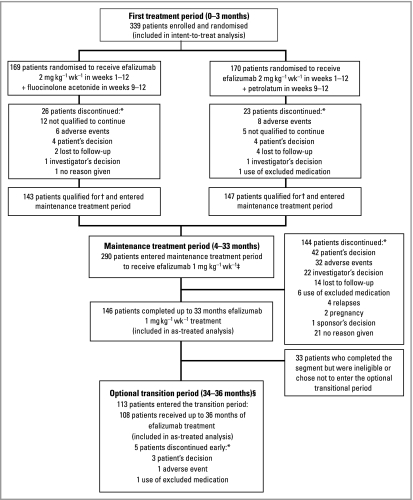
Patient disposition. *Patients were categorized as discontinued only if they withdrew from the study and did not enter follow-up or discontinued from the study segment and entered the follow-up. †Patients who achieved a ≥ 50% improvement in the Psoriasis Area and Severity Index score or static Physician's Global Assessment of mild, minimal or clear at the end of the first treatment period were eligible to enter the maintenance treatment period. ‡Patients who relapsed during months 4–15 ended participation in the current 3-month segment and immediately started the next segment at 2–4 mg kg^−1^ weekly. After month 15, no further dose escalation was permitted. §The optional transition period was made available to patients who had completed 33 months of efalizumab therapy prior to the availability of commercial drug. Other patients transitioned to commercial efalizumab after month 33.

The 3-month first treatment period was completed by 308 patients (90·9%), and 290 (85·5%) patients qualified for and entered into the maintenance treatment period (Fig. 2). A total of 146 patients (43·1%) completed the maintenance treatment period and received 33 months of continuous efalizumab therapy; 113 patients (33·3%) qualified for and entered the subsequent 3-month transition treatment period; and 108 patients (31·9%) received a total of 36 months of efalizumab therapy during the study. The rate of withdrawal during each 3-month segment between month 3 and month 30 ranged from 4·0% to 11·4%.

A total of 231 patients did not complete 36 months of treatment; of these, 33 patients completed 33 months of treatment and were discontinued for administrative reasons (i.e. eligibility for transition to commercial efalizumab or another appropriate therapy at end of study). Of the 198 other patients who discontinued from the study, 61 (30·8%) did so for lack of efficacy, with 17 of these discontinuing at month 3 per protocol. Other reasons for discontinuation were adverse events (47 patients; 23·7%), unknown reasons (22 patients; 11·1%), loss to follow-up (20 patients; 10·1%), and noncompliance, initiation of excluded therapy, pregnancy, withdrawal of consent, relocation or other reasons (48 patients; 24·3%).

### Use of concomitant medication

A total of 64 (18·9%) patients received an excluded systemic therapy that could have affected the patient's psoriasis (Table 1). The majority of these cases involved nonpsoriasis indications; 23 of the patients received concomitant medication for events classified as ‘joint pain’, which included psoriatic arthritis, arthritis, osteoarthritis, gout, knee swelling, tendonitis, bursitis, or pain in the temporomandibular joint, foot, arm or ankle. Most patients who received disallowed systemic therapies were discontinued from the study per study protocol, including 15 patients who received disallowed concomitant medication for joint pain. For efficacy analyses, any such patient remaining in the study was treated using LOCF, taking the last PASI score prior to use of the concomitant systemic therapy. Patients who received an excluded systemic therapy for an indication of psoriasis were included in the efficacy analysis only until the segment in which they first received the excluded medication. These patients were considered nonresponders in that and all subsequent treatment segments. Patients who received a systemic medication for an indication other than psoriasis were evaluated using LOCF, taking the last PASI score prior to use of the concomitant systemic therapy for all subsequent segments.

**Table 1:** Use of excluded concomitant systemic therapies and permitted phototherapies (intent-to-treat population; *n* = 339)

Therapy	Number treated at any time, *n*	Indicated for psoriasis, *n* (%)
Systemic therapy^a^		
Ciclosporin	1	1 (0·3)
Methotrexate	7	2 (0·6)
Systemic retinoids^b^	6	6 (1·8)
Biologics^c^	3	0
Systemic corticosteroids^d^	54	2 (0·6)
Other systemics	2	0
Total number of patients	64	10 (2·9)
Phototherapy^e^	21	21 (6·2)

a None of the systemic therapies listed was permitted per protocol; phototherapy was permitted.

b Bexarotene and acitretin.

c Etanercept.

d Cortisone acetate, dexamethasone, methylprednisolone, prednisolone, prednisone, triamcinolone.

e Phototherapy includes ultraviolet B and Not Otherwise Specified.

During the study, 10 of 339 patients (2·9%) received a concomitant systemic agent for psoriasis: six patients used these for up to 3 months, and four patients used these for up to 6 months. Eight of 10 uses of these agents were during the maintenance period. For efficacy analyses, these patients were treated as nonresponders in all segments beginning with the segment in which the patient received the concomitant treatment.

### Efficacy

**Fig 3:**
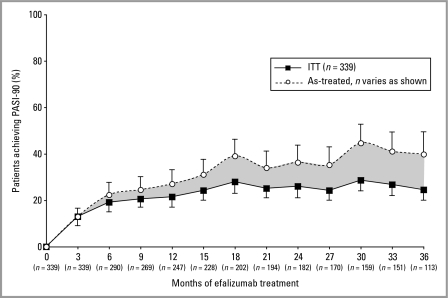
PASI-90 (≥ 90% improvement in the Psoriasis Area and Severity Index score) responses with 95% confidence intervals for the intent-to-treat (ITT) population (*n* = 339) and as-treated population (*n* values listed below axis).

**Fig 4:**
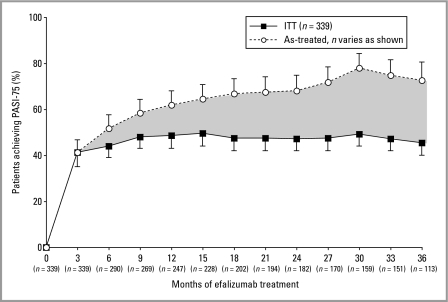
PASI-75 (≥ 75% improvement in the Psoriasis Area and Severity Index score) responses with 95% confidence intervals for the intent-to-treat (ITT) population (*n* = 339) and as-treated population (*n* values listed below axis).

**Fig 5:**
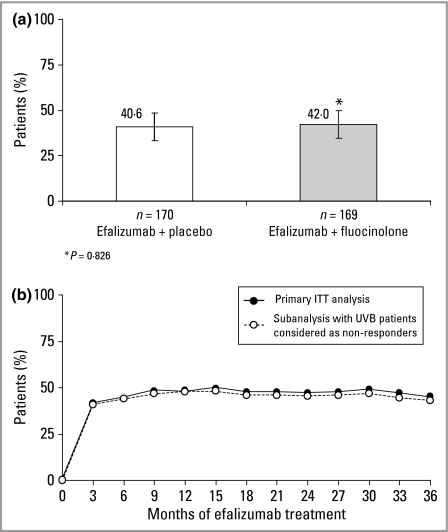
Patients achieving PASI-75 (≥ 75% improvement in the Psoriasis Area and Severity Index score) with or without (a) concomitant topical steroid therapy or (b) ultraviolet (UV) B phototherapy. (a) Intent-to-treat (ITT) analysis of patients using efalizumab with placebo (*n* = 170) or topical fluocinolone (*n* = 169) therapy during the first 3 months of the study. Error bars represent the 95% confidence intervals. (b) ITT analyses in which patients using UVB (*n* = 21) were included in the primary ITT analysis (solid circles) or considered as nonresponders in a subanalysis (open circles) of the results.

The ITT analysis demonstrates the maintenance of efalizumab efficacy throughout 36 months of continuous treatment (Figs 3, 4). As previously reported, after the first 3 months of therapy 82·0% of all patients (*n* = 339) had achieved PASI-50, 41·3% had achieved a ≥ 75% improvement in PASI relative to baseline (PASI-75), and 13·0% had achieved a ≥ 90% improvement in PASI relative to baseline (PASI-90); and 76·1% had received an sPGA rating of mild or better on the OLS scale.^18^ Use of concomitant topical treatment during month 3 demonstrated no significant effect on PASI score, with 42·0% and 40·6% of all patients achieving PASI-75 with fluocinolone acetonide ointment or placebo, respectively (*P* = 0·826; Fig. 5a). After completion of the maintenance treatment period (month 33), response rates for the ITT population were 47·2% PASI-75 and 26·8% PASI-90. After completion of the transition treatment period (month 36), the response rates were 45·4% PASI-75 and 24·5% PASI-90.

Of the 25 patients receiving an escalated dose (3 mg kg^−1^ weekly, *n* = 10; 4 mg kg^−1^ weekly, *n* = 15), PASI-75 was achieved by three patients (two receiving 3 mg kg^−1^ weekly and one receiving 4 mg kg^−1^ weekly).

To assess the effect of concomitant phototherapy on response, a retrospective analysis was performed excluding patients who had used UVB phototherapy. Permitted UVB phototherapy was used by 21 (6·2%) patients during the study (Fig. 5b). In the subanalysis (performed as an ITT analysis with patients using UVB considered as nonresponders), PASI-75 was achieved by 40·7% and 43·1% of patients at 3 and 36 months, respectively, compared with 41·3% and 45·4% achieved by the total population at 3 and 36 months, respectively.

Using the as-treated analysis, after completion of the maintenance treatment period (month 33), response rates were 74·8% PASI-75 (Fig. 4) and 41·1% PASI-90 (*n* = 151) (Fig. 3). After completion of the transition treatment period (month 36), the response rates were 72·6% PASI-75 and 39·8% PASI-90 (*n* = 113). PASI-50 at 33 and 36 months was 94·0%.

**Fig 6:**
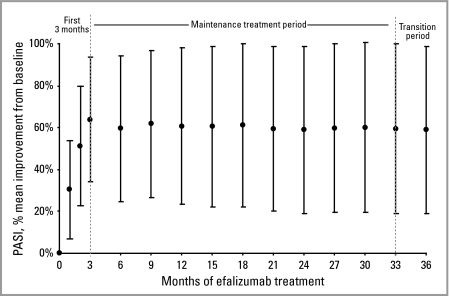
Percentage mean ± SD improvement during each 3-month interval. Values from early termination visits were assigned to the next scheduled visit for Psoriasis Area and Severity Index (PASI) evaluation. Intent-to-treat analyses (*n* = 339) with last observation carried forward (LOCF); baseline was used in LOCF imputation for patients who had no postbaseline visit assessment available.

The positive response of these patients to long-term continuous efalizumab treatment is further illustrated by percentage PASI improvement from baseline: ITT analysis demonstrated a mean improvement of 63·8% by 3 months, a figure that remained stable through 36 months (Fig. 6).

**Fig 7:**
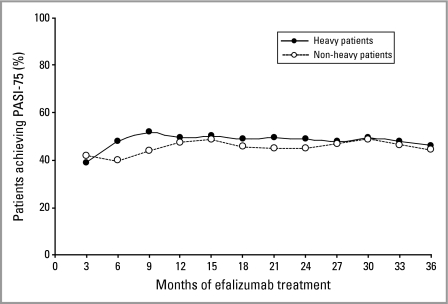
Heavy and nonheavy patient PASI-75 (≥ 75% improvement in the Psoriasis Area and Severity Index score) response to efalizumab over 36 months. Intent-to-treat analysis of response to efalizumab treatment by heavy patients (≥ 91 kg; *n* = 173) and nonheavy patients (< 91 kg; *n* = 166).

Efalizumab was administered to patients in a weight-based dosage throughout the study. The treatment response to efalizumab was maintained for heavy (≥ 91 kg; *n* = 173) and nonheavy (< 91 kg; *n* = 166) patients over the 36-month period (Fig. 7). At 3 months, 39% and 42% of heavy and nonheavy patients, respectively, achieved a PASI-75 response to efalizumab; at 36 months, 46·2% and 44·6%, respectively, achieved PASI-75. Statistical analysis at each point confirmed that the two groups responded similarly.

### Safety and tolerability

Efalizumab was generally well tolerated throughout 36 months of continuous treatment, with no evidence of cumulative or end-organ toxicity. Common adverse events (≥ 5% of patients in any 3-month segment) during long-term treatment included increased cough, rhinitis, sinusitis, and nonspecific infection (e.g. colds). There was no increase over time in the rate of those common adverse events. Likewise, no novel adverse events or increase in the overall incidence of adverse events was noted over time. A low proportion of patients (≤ 3·1%) withdrew due to an adverse event during each 3-month treatment segment of the maintenance period.

#### Infection

**Fig 8:**
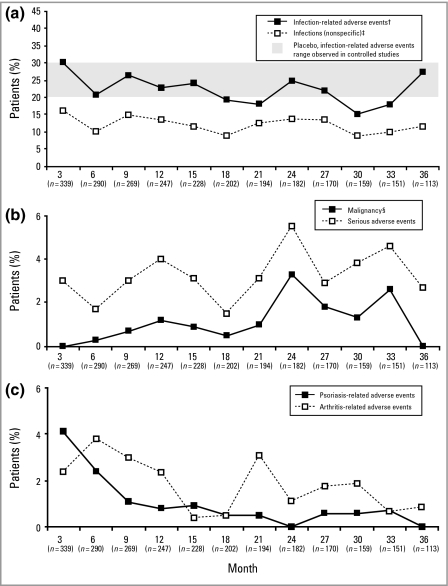
Incidence of selected adverse events (AEs) reported for each 3-month exposure period over 36 months of continuous therapy. Percentages are based on the number of patients and events in each 3-month segment. The graphs show percentages of patients who experienced at least one AE of the type specified during any 3-month period; multiple occurrences of the same event for a patient were counted once in the overall incidence. (a) Infection-related AEs/infections (nonspecific). The shaded region indicates the range of infection-related AEs observed for patients receiving placebo for 3 months in controlled studies. (b) Serious AEs/malignancies. (c) Psoriasis-related AEs and arthritis-related AEs. †Infection-related AEs include AEs that were diagnoses of infection or strongly suggestive of infection. ‡Nonspecific infections, mostly upper respiratory tract infections (e.g. bronchitis, pharyngitis, rhinitis and sinusitis), were a subset of infection-related AEs. §Skin cancer: a subset of serious AEs.

The 30·2% incidence of infection-related adverse events (adverse events that were diagnoses of infection or strongly suggestive of infection) during the first 3-month treatment segment was comparable with that observed during previously reported 3-month placebo-controlled efalizumab clinical trials^14^,^15^,^23^ and did not increase over the course of the study (Fig. 8a).

### Malignancy

Malignancy rates were low and stable throughout the study (Fig. 8b, Table 2). Most malignancies reported during the study were nonmelanoma skin cancers (NMSCs) (22 events in 11 patients), with prior phototherapy [psoralen + UVA (PUVA) and/or UVB] or extensive sun exposure being reported by eight of these patients. Lymphoma was noted in two patients (one after 24 months and one after 30 months of treatment), gastrointestinal carcinoma in two patients (one after 12 months and one after 30 months of treatment), lung carcinoma in one patient after 24 months of treatment, prostatic carcinoma in one patient after 27 months of treatment, and cutaneous melanoma *in situ* in one patient after 6 months of treatment.

**Table 2:** Malignancies reported by study segment

Segment of study	Type of malignancy	Events reported (*n*)
4–6 months	Nonmelanoma skin carcinoma	1
7–9 months	Nonmelanoma skin carcinoma	1
	Malignant melanoma	1
10–12 months	Nonmelanoma skin carcinoma	3
13–15 months	Nonmelanoma skin carcinoma	1
	Gastrointestinal carcinoma	1
16–18 months	Nonmelanoma skin carcinoma	2
19–21 months	Nonmelanoma skin carcinoma	2
22–24 months	Nonmelanoma skin carcinoma	7
25–27 months	Lymphoma-like reaction	1
	Carcinoma of lung	1
	Nonmelanoma skin carcinoma	1
28–30 months	Nonmelanoma skin carcinoma	2
	Prostatic carcinoma	1
31–33 months	Lymphoma-like reaction	1
	Gastrointestinal carcinoma	1
	Nonmelanoma skin carcinoma	2
Total, 0–36 months		29

#### Psoriasis-related adverse events

Psoriasis-related adverse events were noted in 14 of 339 (4·1%) patients in the first 3-month segment, and in seven of 290 (2·4%) patients during the second 3-month segment (Fig. 8c). Eight of 14 patients and seven of seven patients during the first and second 3-month segments, respectively, experienced a recurrence of mild or moderate plaque psoriasis. However, throughout the 36-month study, events of psoriasis flare with atypical papular or plaque lesions (*n* = 5), psoriatic erythroderma (*n* = 3), inverse psoriasis (*n* = 2), guttate psoriasis (*n* = 2) and pustular psoriasis (*n* = 1) were also reported. The incidence of patients experiencing these events decreased to less than 1% over the course of the 36 months of the study.

### Arthritis

Arthritis-related adverse events, including events of arthritis, psoriatic arthritis and osteoarthritis, were experienced by 45 patients over the 36-month period of the study; 16 of these patients reported new onset of psoriatic arthritis. Eighteen of the 45 patients withdrew from the study, including 15 patients who were treated with disallowed systemic agents for management of their arthritis symptoms. The arthritis symptoms of the remaining 27 patients were managed with nonsteroidal anti-inflammatory drugs. Six patients (1·8%) experienced a serious arthritis adverse event during the study: three events were observed during the first 3-month treatment segment, and the other three events occurred during the subsequent 33 months (Fig. 8c).

#### Other adverse events

There were 13 anaemia-related adverse events, all nonserious, reported in seven patients: eight events of anaemia in six patients, one event of iron-deficiency anaemia in one patient, and four events of hypochromic anaemia in two patients. There were no incidences of haemolytic anaemia. Six adverse events of thrombocytopenia were reported in five patients, three of whom were discontinued. Two of the events were considered serious. One patient developed a serious event of thrombocytopenia approximately 17 months after initiating efalizumab. He was hospitalized for dehydration with a platelet count of 33 × 10^9^ L^−1^ and treated with prednisone; 7 days later his count was 106 × 10^9^ L^−1^. Prednisone was tapered and the patient was discontinued from the study. The second patient developed a serious event of thrombocytopenia after approximately 33 months of treatment with efalizumab. The platelet count at presentation was 16 × 10^9^ L^−1^; dexamethasone was prescribed for 5 days and the patient was removed from the study. The event resolved with a rapid recovery of the patient's platelet count. One patient experienced two mild events of thrombocytopenia and was removed from the study after the second event with a platelet count of 80 × 10^9^ L^−1^. This patient had a platelet level below the normal range (< 130 × 10^9^ L^−1^) at screening and throughout the study.

One patient developed congestive heart failure in the 31–33-month segment of the study, an event deemed unrelated to the study drug by the investigator.

The rate of withdrawal for any reason during each 3-month segment between month 3 and month 30 was low, ranging between 4·0% and 11·4%. This rate is similar to that observed in other efalizumab studies with 3-month, placebo-controlled treatment periods.^13^,^14^

## Discussion

This report presents final results from the longest continuous-treatment prospective study of a biologic therapy for psoriasis. Clinical improvements, as measured by PASI-75 response rates observed after the first 3 months of efalizumab therapy in patients with moderate-to-severe chronic plaque psoriasis, were maintained throughout 36 months of continuous dosing. The use of concomitant UVB phototherapy or topical treatment had no additive effect on the outcome of response in these patients.

For long-term studies, ITT analysis underestimates the actual benefit; thus, we have conducted an additional as-treated analysis as recommended in the revised Consolidated Standards of Reporting Clinical Trials (CONSORT) statement.^24^ This additional analysis is intended to complement the primary ITT analysis and to address the lack of any globally applicable means of handling missing values, as recognized by the ICH and CPMP.^21^ As-treated analysis considers only the patients who remain on therapy and does not take into consideration those who discontinued treatment due to an adverse event, lack of efficacy or any other reasons.

During the first 3 months of the study, 14·5% of the patients discontinued; this rate included patients who did not meet the eligibility criteria to continue and were forced to withdraw from the study (17 patients; 5·0%). Between months 4 and 6 of the study, 7·2% discontinued. These withdrawal rates are comparable with those observed in previously published extended phase III trials of efalizumab^15^,^16^ and compare favourably with the withdrawal rate of 28% observed in a 4-month trial of methotrexate treatment of 44 patients with psoriasis.^25^ The rates of withdrawal continued to be low and consistent in each 3-month segment of the study, ranging from 4% to 11·4%, except for the 31–33-month segment, when the rate rose to 25·2% due to the study restriction that patients were ineligible to enter the 34–36-month segment if efalizumab was available at the time of completion of the 31–33-month segment. Overall, 18·0% of patients withdrew because of lack of efficacy or worsening of psoriasis.

Efalizumab was generally well tolerated during up to 36 months of continuous treatment. There was no increase over time in the overall incidence of adverse events, no increase in common adverse events, no emergence of new adverse events, no apparent trend towards an increase in the incidence of clinically significant adverse events, and no evidence of cumulative or end-organ toxicity. Clinical trials and postmarketing reports have infrequently noted new-onset or worsening arthritis.^26^ Arthritis-related adverse events remained low, ranging between 0·4% and 3·8% throughout the 36-month study. This corroborates the results of a pooled analysis of adverse events related to arthropathy from five clinical trials of efalizumab in patients with psoriasis.^27^

During the study, events of thrombocytopenia were noted in five patients; low platelet counts and thrombocytopenia have been observed in postmarket monitoring.^26^ Thus, physicians should follow patients closely for signs and symptoms of thrombocytopenia with regular assessment of platelet counts.

Two lymphomas (appearing after 24 and 30 months of treatment), one melanoma (appearing after 6 months of treatment), 22 cases of NMSC in 11 patients and five events of solid tumour (including the one case of melanoma) were reported during this 36-month study. An increased frequency of lymphoproliferative diseases^28^–^30^ has been observed among patients with psoriasis compared with the general population. In addition, patients with psoriasis appear to be at an increased risk for the development of skin cancers,^31^,^32^ possibly associated with the prior use of PUVA.^8^ Indeed, eight of the 11 patients with NMSC had been treated previously with phototherapy or reported extensive sun exposure. The relatively small size of the cohort limits the conclusions that can be drawn regarding rare events such as cancers; however, a separate analysis of malignancy rates, based on 14 pooled efalizumab clinical trials, has been performed to address this question.^33^ The incidence rate for solid tumours was 0·47/100 patient-years in efalizumab-treated patients and 0·54/100 patient-years in placebo-treated patients, and it ranged between 0·26/100 and 0·44/100 patient-years in three external reference databases.^33^ In the 36-month study reported here, solid tumour events occurred at a rate of 0·49/100 patient-years. Further analysis of the effect of efalizumab on malignancy rates will be possible through the Raptiva Epidemiologic Study of Psoriasis Outcomes and Safety Events (RESPONSE), an observational patient registry recording safety for up to 5 years for efalizumab-treated (*n* = 5000) and other biologic-treated (*n* = 2500) patients. Because patients with internal malignancies and melanoma were excluded from the trials, caution should be exercised when considering the use of efalizumab in patients at high risk for malignancy or with a history of malignancy.

No evidence of opportunistic infections (e.g. tuberculosis reactivation), significant increase in infections, or neurological disorders (including multiple sclerosis) was observed in this long-term study of efalizumab therapy. Congestive heart failure was noted in a single patient. A postmarketing study is also being conducted to assess the effect of long-term exposure to efalizumab on the incidence of serious adverse events.

This is the longest study of any biologic therapy for psoriasis. Moreover, the use of systemic concomitant medications for psoriasis-related and nonpsoriasis-related events was accounted for in this analysis, with efficacy results similar to those published from an interim analysis^18^,^19^ in which the use of concomitant medications was not accounted for, thus suggesting that the use of concomitant medications did not greatly affect efficacy.

Based on the clinical and safety data obtained in this 3-year clinical study, continuous efalizumab therapy represents an appropriate treatment for long-term therapy for many patients with plaque psoriasis.
